# Application of yeast in plant-derived aroma formation from cigar filler leaves

**DOI:** 10.3389/fbioe.2022.1093755

**Published:** 2022-12-21

**Authors:** Lan Yao, Chenyi Huang, Jingyi Ding, Tongtong Zhang, Jun Yu, Chunlei Yang, Xiong Chen

**Affiliations:** ^1^ Key Laboratory of Fermentation Engineering (Ministry of Education), Cooperative Innovation Center of Industrial Fermentation (Ministry of Education & Hubei Province), HBUT National “111” Center for Cellular Regulation and Molecular Pharmaceutics, College of Bioengineering and Food, Hubei University of Technology, Wuhan, China; ^2^ Hubei Institute of Tobacco Science, Wuhan, China

**Keywords:** plant-derived aroma, cigar filler leaves, aroma-producing yeast, solid-state fermentation, olfactory threshold, aroma characteristics

## Abstract

**Introduction:** There are various degrees of defects of cigar filler leaves after air drying.

**Methods:** In order to improve the quality and plant-derived aroma content of cigar filler leaves, nine aroma-producing yeasts were applied in artificially solid-state fermentation of cigar filler leaves in this study. The differences with various yeasts application were compared by chemical composition and GC-MS analysis.

**Results and discussion:** The results showed that 120 volatile components were identified and quantified in cigar filler leaves after fermentation, including aldehydes (25 types), alcohols (24 types), ketones (20 types), esters (11 types), hydrocarbons (12 types), acids (4 types) and other substances (23 types). Based on the analysis of odor activity value (OAV), the OVA of fruity and floral aroma components were higher. It was found that floral aroma are the representative aroma types of cigar filler leaves treated with *Clavispora lusitaniae*, *Cyberlindera fabianii*, *Saccharomycosis fibuligera* an*d Zygosaccharomyces bailii* R6. After being inoculated with *Hanseniaspora uvarum* J1, *Hanseniaspora uvarum* J4 and *Pichia pastoris* P3, the OAV of fruity aroma in cigar filler leaves was the highest, followed by tobacco aroma and woody aroma. The correlation between volatile components of cigar filler leaves with different yeasts was revealed after PCA analysis. It was concluded that the quality of cigar filler leaves was improved, and cigar filler leaves fermented with different yeasts showed different flavor.

## 1 Introduction

Cigar is composed by three parts, cigar wrapper, cigar binder and cigar filler (Li et al., 2015). Cigar wrapper is the clothes of cigars, which can be observed directly, and is a tool to protect and beautify cigars. Cigar Binder is the part covered by the cigar wrapper, which is used to tightly wrap the cigar filler to form the shape of the cigar. Cigar Filler is the most internal structure of a cigar, which accounts for about 75% of the weight of the whole cigar. Cigar Filler determines the style, quality, and the flavor of a cigar during smoking (Chen et al., 2019).

Cigar leaves can’t be directly used after being dried. To meet the needs of industrial processing, fermentation process is a necessity. There are many macromolecular substances in cigars leaves, which will produce a lot of unpleasant gases during combustion, affecting the overall quality of cigars. After fermentation, the qualities of tobacco are improved by eliminating harmful odors, degrading harmful substances, reducing offensive odor, and producing tobacco-specific flavors (Yang et al., 2018; Li J et al., 2020).

The mechanism of fermentation mainly includes enzymatic, microbial and chemical reactions (Zhang L et al., 2021). Microbiology plays an important role in the process of fermentation and aging of cigar tobacco leaves. Microbes on the surface of cigar tobacco leaves can synthesize and secrete a variety of extracellular hydrolase, which can accelerate the degradation of starch, pectin, cellulose, protein, lignin and other macromolecular substances into some small molecular compounds to form aroma compounds. (Xu et al., 2021; Zhang et al., 2022).

There are many microorganisms in nature that can produce aroma substances, which provide more ways to develop new natural flavors. Aroma-producing yeasts are a class of yeasts that can produce aroma substances and are now widely used in the food industry (Zhang et al., 2015). In recent years, with the rapid development of microbial technology, more and more scholars pay attention to the screening and application of microorganisms that can enhance the quality of tobacco. It has been proved that fermentation with aroma-producing microorganisms to improve the aroma quality of tobacco is a feasible method.

A strain of *Pichia terricola* MG6 was screened and isolated from the epidermis of grapes (Yan et al., 2021). The fermentation broth was analyzed by GC-MS technique, and results showed that the content of aroma components such as ketones, alcohols and esters was increased by different levels. Furthermore, the tobacco flavors prepared from the fermentation broth of this strain did improve the quality of cigarettes. *Hanseniaspora* strain YG-4 isolated by Guo et al. (2019) proved that it could produce a distinct floral and sweet aroma in the fermentation broth. And the fermentation broth prepared using this strain with tobacco leaf powder showed an increased content of 2-phenylethanol, phenylethyl benzoate, 2-pentenoic acid and 1-phenyl-3-aminopyrazole compared with the control group. A sweet aroma producing strain of bacteria named Lizhi-01 was isolated and purified from fresh litchi and it was found that the aroma components of tobacco leaves after fermentation with this strain were mainly 2-phenylethanol, 2,3-butanediol, furfural and Palmitic acid (Zhou et al., 2010).

The odor activity value (OAV) refers to the ratio of the concentration of an aroma component in the aroma system to its threshold value. It can be used as an indicator of the sensory impact of each volatile compound on the overall aroma (Peinado et al., 2004). Generally, components with OAV >1 are called modified aroma components, components with OAV >10 are considered as key aroma components. And a higher OAV value indicates a greater contribution of the component to the overall aroma (Pino and Queris., 2011).

Raw and lower grade tobacco leaves are often with more impurities, less delicate smoke, less mellow flavor, and even bitterness, pungency, and astringency. Therefore, fermentation is needed to improve the quality of raw tobacco leaves. The fermentation process of cigar tobacco is actually a biochemical reaction process that involves the interaction of substrate with microorganisms and enzymes (Niu et al., 2020). Microorganisms can be added artificially during the processing process to shorten the fermentation time and improve the quality of the cigar leaves including the desired color, aroma and combustion properties. Theoretically, the fermentation of cigar leaves with the addition of microorganisms with known characteristics can result in a more distinctive cigar product. At present, domestic cigar raw materials lack distinguished aroma style, superior aroma quantity and quality. Thus, improving the characteristics of domestic cigar is a crucial step in the development of cigar industry. Many studies have shown that yeast, bacteria as well as fungi can produce aroma. The application of *Bacillus* in tobacco fermentation has been reported frequently, while there are limited reports on the application of aroma producing yeast in cigar filler leaves fermentation so far.

In this paper, nine aroma-producing yeasts were applied in the solid fermentation of cigar filler leaves, among which C*lavispora lusitaniae* can produce alcohols and esters (Wang et al., 2022). *Saccharomyces cerevisiae* has the ability to produce acid and esters (Minebois et al., 2020; Yang et al., 2022). *Cyberlindnera fabianii* could generate complex aromas during the brewing process, whose liquid culture has a better ability to produce ethyl acetate (Van Rijswijck et al., 2019). *ZygoSaccharomyces rouxii* can grow in high salt and high temperature environments, and its main role in the fermentation process is to ferment alcohols and synthesize a variety of ester aroma components, glycerol and polyols, as well as other ketones and phenols (Escott et al., 2018; Dai et al., 2020; Niu et al., 2022). *Saccharomycopsis fibuligera* is applied in various koji. It can produce amylase, glycosylase, and esterase, and has a positive effect on the production of volatile compounds (Lee et al., 2018; Yeong et al., 2018; Ma C et al., 2022). *Hanseniaspora uvarum* can produce volatile acids, organic acids, aldehydes, alcohols and other secondary metabolites (Hu K et al., 2018; Wei et al., 2020). *Pichia pastoris* is capable of producing esters such as ethyl acetate, isobutyl acetate and isoamyl acetate (Wei et al., 2020; Jia et al., 2021). *Zygosaccharomyces bailii* forms more alcohols, acids, esters and aldehydes (Xu C. P et al., 2018).

After fermentation, the total aroma and OAV value of cigar tobacco leaves inoculated with various strains of aroma-producing yeast were analyzed and compared. Strains that contribute to the unique aroma characteristics of cigar were screened out. This may also provide reference for the further application of bio-fermentation technology to produce cigar with different aromas.

## 2 Materials and methods

### 2.1 Materials

The cigar filler leaves were CX-014 after air drying in Enshi, Hubei Province, China. All the chemical reagents used in the study were purchased from Sinopharm group, China.

*Saccharomyces cerevisiae*, *ZygoSaccharomyces rouxii*, *Clavispora lusitaniae*, *Saccharomycopsis fibuligera*, *Cyberlindnera fabianii*, *Hanseniaspora uvarum* J1, *Hanseniaspora uvarum* J4, *Pichia pastoris* P3 and *Zygosaccharomyces bailii* R6 are all preserved strains in our own laboratory.

Yeast extract peptone dextrose (YEPD) medium, yeast extract powder 10 g/L, glucose 20 g/L, peptone 20 g/L, solid medium with agar 20 g/L.

### 2.2 Experimental methods

#### 2.2.1 Preparation of yeast seed solution

Yeasts stored in −80°C refrigerator were transferred to liquid YEPD medium and incubated at 30°C and 200 r/min for 24 h.

The obtained seed solution was centrifuged at 4°C and 12000 r/min for 5 min, the supernatant was discarded to collect wet cells, and then resuspended in sterile deionized water. The operation was repeated twice to obtain wet cells without fermentation broth. Then sterile YEPD medium was applied to dilute the yeast cell concentration to 10^8^ cfu/ml.

#### 2.2.2 Solid-state fermentation of cigar filler leaves

Referring to the method described earlier (Qin et al., 2020), the obtained yeast cell suspension (10% ml/g, based on the ratio of volume to dry mass of cigar filler leaves) was sprayed on the surface of cigar filler leaves. The control groups were sprayed with sterile water and liquid YEPD medium, respectively. The moisture content of the cigar filler leaves was controlled at 30%. After the moisture was balanced, the cigar filler leaves were put into a sealed bag and placed in a cabinet with constant temperature and humidity (temperature 30°C, humidity 80%) to ferment for 7 days.

#### 2.2.3 Determination of conventional chemical composition of tobacco

Main chemical components in cigar filler leaves, including total sugar, reducing sugar, nicotine, total nitrogen, potassium and chlorine were evaluated by continuous flow analytical system, based on the standard of tobacco industry (YC/T 159-2002, 2002; YC/T 160-2002, 2002; YC/T 161-2002, 2002; YC/T 162-2011, 2011; YC/T 217-2007, 2007). The quantification results were based on dry weight of cigar filler leaves.

#### 2.2.4 Determination of volatile aroma components

The samples were processed using simultaneous distillation extraction (SDE) technique and analyzed for aroma substances by chromatography-mass spectrometry (GC-MS) (Yao et al., 2022). Briefly, after drying and wiley-milled (screen size <2 mm was used), 10 g samples were applied for aroma components extraction. Then, saturated NaCl and dichloromethane were applied as the extraction solvents. After extraction, the extract were collected and concentrated to 2 ml, with 50 μl 1.2028 mg/ml phenylethyl acetate as internal standard.

#### 2.2.5 Data processing

Chromatograms were analyzed using the GCMS solutionver. 4.11 (Agilent Technologies Inc, United States). The mass spectra data were compared with spectra in the NIST reference library (NIST14) of the GC/MS data system for identification of volatile compounds. Origin 9 (Origin Lab, Massachusetts, United States) was applied to draw the histogram. PCA analysis was performed with SIMCA-P 14.1 (Umetrics, Malmo, Sverige), and cluster heat map was drawn with TBtools (Guang zhou, China).

## 3 Results and analysis

### 3.1 Chemical composition analysis

**FIGURE 1:**
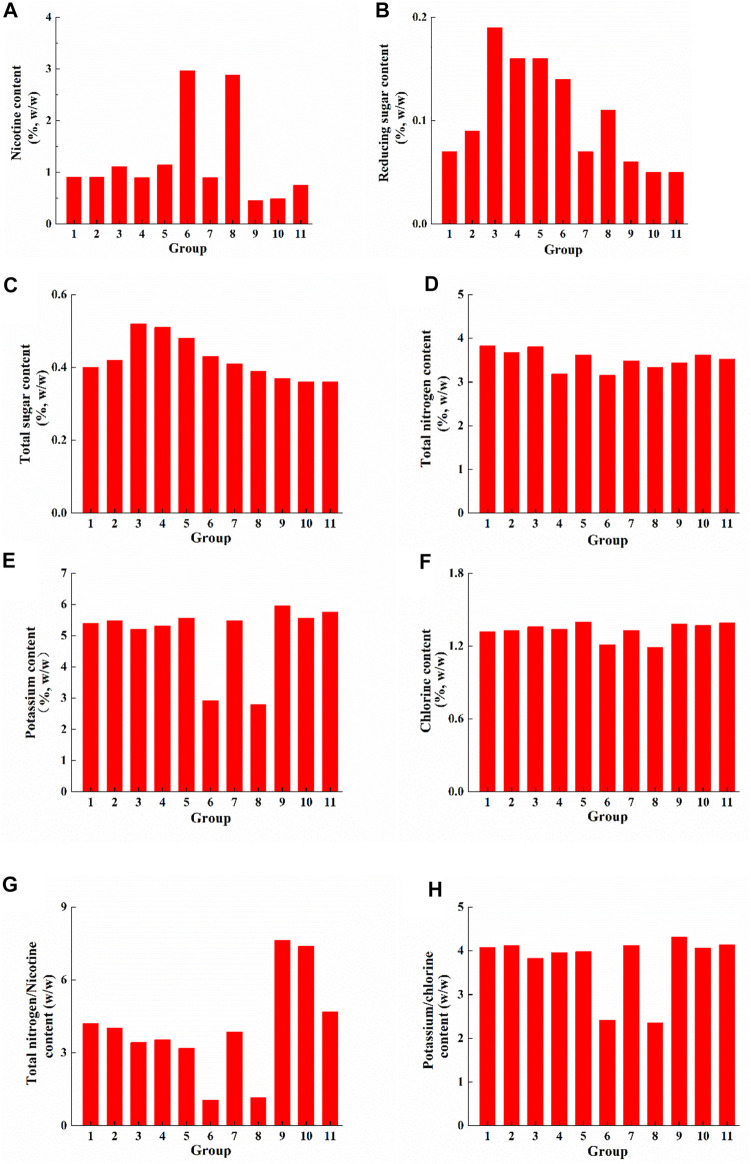
Chemical components analysis of cigar filler leaves after fermentation. **(A)** Nicotine content changes; **(B)** Reducing sugar content changes; **(C)** Total sugar content changes; **(D)** Total nitrogen content changes; **(E)** Potassium content changes; **(F)** Chlorine content changes. Note: 1-Water; 2-Medium; 3-Medium + *Clavispora lusitaniae*; 4-Medium + *Saccharomyces cerevisiae*; 5-Medium + *Cyberlindnera fabianii* 6-Medium + *ZygoSaccharomyces rouxii*; 7-Medium + *Saccharomycopsis fibuligera*; 8-Medium + *Hanseniaspora uvarum* J1; 9-Medium + *Hanseniaspora uvarum* J4; 10-medium + *Pichia pastoris* P3; 11-medium + *Zygosaccharomyces bailii* R6.

The main chemical components of tobacco leaves can affect the smoke characteristics to some extent and can be used as an indicator to identify the quality of tobacco leave (Gao et al., 2011). The contents of nicotine, reducing and total sugars, potassium, and chloride in fermented cigar filler leaves were determined, and the results were shown in Figure 1. It was showed that the nicotine content of cigar filler leaves after fermentation with the addition of *Clavispora lusitaniae*, *Cyberlindnera fabianii*, *ZygoSaccharomyces rouxii* and *Hanseniaspora uvarum* J1 was higher than that of the control group (Figure 1A). The highest nicotine content was found in the group with the addition of *ZygoSaccharomyces rouxii* (2.99%), followed by the group with the addition of *Hanseniaspora uvarum* J1 (2.88%). The nicotine content of other treatment group decreased to various degrees. The increase in relative nicotine content in some group may be caused by the degradation of other substances, such as starch, protein and cellulose.

Cigar leaves contain very low sugar content (basically below 0.5%), because sugar is almost consumed after drying. Among them, groups treated with *Clavispora lusitaniae*, *Saccharomyces cerevisiae*, and *Cyberlindnera fabianii* showed the highest reducing and total sugar content (Figures 1B,C). Probably because these three yeast strains have the ability to produce α-amylase and glycosylase (Meng and Zhang, 2021), which can degrade the starch in the cigar filler leaves. The reducing and total sugar contents of the other groups were not significantly different compared with the control groups.

The total nitrogen content of high-quality cigar tobacco is generally around 4%–6%, and the ratio of nitrogen to nicotine is between 3 and 4 (Wu et al., 1999). When the total nitrogen content is lower, the taste is insipid. The smoke generated by high total nitrogen content is strong, pungent and irritating (Liu, et al., 2022). Except for nicotine, nitrogenous compounds in cigar leaves include proteins, amino acids, and amide compounds, can often make the smoke bitter and rough (Yang C et at., 2018). The total nitrogen content decreased in all the groups with the addition of yeast (Figure 1D). Among them, the nitrogen-nicotine ratio was less than 3 in the treatment groups with the addition of *ZygoSaccharomyces rouxii* and *Hanseniaspora uvarum* J1, while more than 4 in the treatment groups with the addition of *Hanseniaspora uvarum* J4, *Pichia pastoris* P3, and *Zygosaccharomyces baili* R6 (Figure 1G). Tobacco with a potassium-chloride ratio above 4 show better combustibility, while below 2 is prone to flameout (Yin et al., 2018). The treatment groups with *ZygoSaccharomyces rouxii* and *Hanseniaspora uvarum* J1 had low potassium-chlorine ratios and therefore poor combustibility, while the other groups had better combustibility with potassium-chlorine ratios around 4 (Figure 1H).

### 3.2 Analysis of the types and contents of volatile substances in cigar filler leaves after addition with different aroma-producing yeasts

**FIGURE 2:**
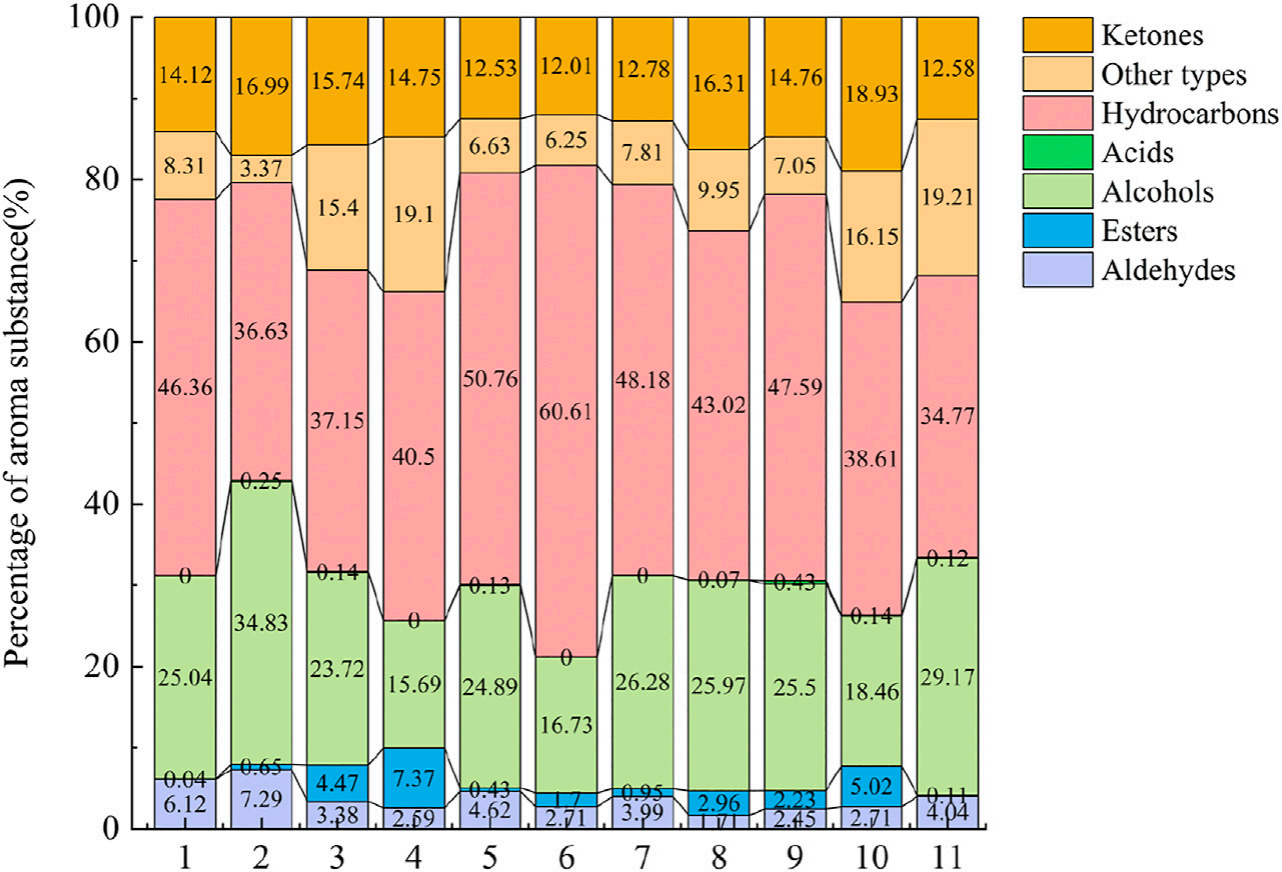
Comparison of types of volatile component in cigar filler leaves after fermentation with different yeast. Note: The numbers in the horizontal coordinate represent the same groups as in Figure 1.

Aroma composition is one of the most important indicators to evaluate the quality of cigar. Aroma in tobacco is determined by the composition and ratio of aroma-causing substances. In order to analyze the effects of different aroma-producing yeasts on the types and contents of volatile substances in cigar filler leaves, volatile compounds were identified by GC-MS on the fermented filler leaves, and the results were shown in Figure 2. Volatile components can be classified into seven categories: aldehydes, alcohols, ketones, esters, hydrocarbons, acids, and others. The quantity and relative content of volatile substances in cigar filler leaves were various after different treatments with yeast. Among them, the amount of aldehydes (25 types) ranked the first, followed by alcohols (24 types), ketones (20 types), esters (11 types), hydrocarbons (12 types), acids (4 types), and others (23 types). The total amount of volatile components after yeast treatment ranged from 311.68 to 637.38 μg/g. And group treated with *Cyberlindnera fabianii* showed the highest total amount of volatile components. More details were shown in SupplementaryTable S1.

Ketones accounted for 12.01%–18.93% of the total aroma, which were mainly cembranoids and carotenoids degradation products. Relatively high content of ketones were solanone, perhydrofarnesyl acetone and farnesyl acetone. Solanone has a fresh carrot aroma and can make the smoke rich, mellow and delicate (Yun, 2016). Its highest content reached 22.69 μg/g and 24.14 μg/g in the *Cyberlindnera fabianii* and *Saccharomycopsis fibuligera* treatment groups, respectively. In terms of aroma composition after different aroma-producing yeast treatments, *Cyberlindnera fabianii* (79.88 μg/g) and *Hanseniaspora uvarum* J1 (78.28 μg/g) treatments showed the highest total amount of ketones in the cigar filler leaves.

Aldehydes that mainly from phenylalanine metabolites were dominantly comprised by Α,2,6,6-tetramethyl-1-cyclohexene-1-crotonaldehyde, 3-(2,6,6-trimethyl-1-cyclohexen-1-yl)acrylaldehyde, benzaldehyde and (Z)-7-hexadecenal, which took up for 1.71%–7.29% of the total aroma. Α,2,6,6-tetramethyl-1-cyclohexene-1-crotonaldehyde is one of the synthesis products of β-ionone and ethyl chloroacetate (Deng et al., 2021), showing tobacco and nutty aroma. 3-(2,6,6-trimethyl-1-cyclohexen-1-yl)acrylaldehyde is one of the dominant components of the smoke. Benzaldehyde is one of the metabolites of phenylalanine and has a strong almond and cherry flavor, as well as a slight woody aroma (Liu et al., 2019). Compared with other groups, cigar filler leaves innocolated with *Cyberlindnera fabianii* (29.43 μg/g) and *Saccharomycopsis fibuligera* (22.54 μg/g) had higher levels of aldehydes than the other groups.

Alcohols that mainly derived from chlorophyll degradation products, phenylalanine degradation products and maillard reaction products occupy 15.69%–34.83% of the total aroma. Among them, phytol was the predominating alcohols, followed by furfuryl alcohol, 2-phenylethanol and benzyl alcohol. Phytol is converted from the decomposition of neophytadiene, which has a delicate aroma (Wu et al., 2019). Furfuryl alcohol is a kind of Maillard reaction product (Wu et al., 2019) and is one of the main contributing components of the burnt flavor. Benzyl alcohol and 2-phenylethanol are phenylalanine metabolites, which shows rosy aroma (Wu et al., 2019). The alcohol content was relatively high after fermentation with the addition of *ZygoSaccharomyces rouxii* (158.66 μg/g) and *Saccharomycopsis fibuligera* (148.30 μg/g), followed by the treatment groups with *Hanseniaspora uvarum* J4 (130.85 μg/g) and *Hanseniaspora uvarum* J1 (124.63 μg/g), while the control groups only contained 81.66 µg/g and 103.09 μg/g of alcohols, respectively.

Esters that mainly derived from amino acid and fatty acid metabolites accounted for 0.04%–7.37% of the total aroma (Shi et al., 2019). The highest ester content was found in the group treated with *Saccharomyces cerevisiae* (32.00 ug/g) and *Pichia pastoris* P3 (20.08 μg/g), followed by *Hanseniaspora uvarum* J1 (14.22 μg/g), *Clavispora lusitaniae* (13.93 μg/g), and *Hanseniaspora uvarum* J4 (11.42 μg/g). The ester content in the control groups was only 0.12 μg/g (water) and 1.91 μg/g (YEPD medium), respectively, indicating a greater contribution of aroma-producing yeast to the production of esters.

Hydrocarbons that mainly consist of neophytadiene, dipentene dioxide and (+)-Limonene take up for 34.77%–60.61% of the total aroma compounds. Neophytadiene, a kind of chlorophyll degradation products (Wu et al., 2019), is the most abundant component of volatile substances in cigar. It reduces the irritation of the smoke and makes it soft and pleasant, and also could help other volatile aroma substances, aroma-causing substances and added aroma components to enter the smoke (Wang et al., 2015). Among all treatments, the highest levels of neophytadiene were found in the group added with *Cyberlindnera fabianii* (323.54 μg/g) and *Saccharomycopsis fibuligera* (271.92 μg/g), followed by *Hanseniaspora uvarum* J4 (244.16 μg/g) and *ZygoSaccharomyces rouxii* (216.58 μg/g), while the neophytadiene levels in the control group sprayed with sterile water and YEPD medium were 149.7 μg/g and 107.04 μg/g, respectively. Dipentene dioxide showed a menthol aroma and was highest in the group supplemented with *Cyberlindnera fabianii* and *Hanseniaspora uvarum* J4. (+)-Limonene had a pleasant fresh orange aroma and was only present in group supplemented with *Hanseniaspora uvarum* J4 (0.31 μg/g) and *Pichia pastoris* P3 (0.53 μg/g).

The substances in the other classes were mainly products of the Maillard reaction, e.g., pyridines and furans. 3-acetylpyridine showed sweet nut, hawthorn and popcorn aromas and its content was the highest after the addition of *Hanseniaspora uvarum* J4 (8.57 μg/g) and *Pichia pastoris* P3 (8.53 μg/g).

### 3.3 Analysis of the contribution of aroma components to cigar filler leaves

The OAV value can be used to determine the contribution of aroma components to the aroma system in two dimensions: concentration and threshold. Components with OAV <1 are usually considered as potential aroma substances, while aroma components with OAV >1 are considered to have a certain degree of contribution to the overall aroma. The larger their OAV value, the greater of their contribution (Greger and Schieberi, 2007). The OAV results of each aroma component of the cigar leaves were shown in Table S2. 24 aroma compounds of OAV >1 were furfuryl alcohol, 4-hydroxy-3-methoxystyrene, 6-methylhept-5-en-2-one, β-cyclocitral, (+)-Limonene, 5,9-dimethyl-deca-4,8-dienal, 2-hexenal, β-damascone, dihydrodamascenone, damascenone, citronellal, 2-phenylethanol, indole, benzyl alcohol, benzaldehyde, (2E,4E)-2,4-Nonadienal, phenylacetaldehyde, geranyl acetone, 4-ketoisophorone, isophorone, 4,7,9-megastigmatrien-3-one, 3-acetylpyridine, hexanal, and L-menthol. However, the contribution of these substances to cigar aroma was various. Although the OAV values of the key aroma compounds in the samples varied, the OAV values of 2-hexenal, β-damascone, dihydrodamascenone, and damascenone were much higher than those of the other aroma compounds in all samples. It is speculated that they play key roles in the aroma of cigars.

β-damascone, dihydrodamascenone, and damascenone contributed more to the floral aroma. The OAV of the three representative substances of baking fragrances were relatively low. Perhaps baking fragrances are not the most prominent characteristic aroma of cigars, but rather serve as auxiliary fragrances to make cigar smoke more harmonious. 5,9-dimethyl-4,8-decadienal and 2-hexenal have much higher OAV values than the other five aroma components, playing a more important role in demonstrating the fruity aroma. Among the 3 components of woody aroma, isophorone and 4-ketoisophorone had low OAV values, and were identified as a modifying aroma substance of cigar. 2-Hexanal had higher OAV values and was the main contributing component of woody aroma. The content of 2 herbal flavor was low, the OAV values of L-menthol were greater than 1, which was more important for the medicinal aroma.

After calculating the odor activity values of the aroma-causing components, they were categorized and summarized according to the flavors provided. And the flavor intensity of cigar leaves after different yeast treatments were compared, as shown in Table S2. The odor activity values were floral > fruity > tobacco > woody > roasted > herbal after addition with *Clavispora lusitaniae*, *Cyberlindnera fabianii and Saccharomycopsis fibuligera*. *Saccharomyces cerevisiae* and *ZygoSaccharomyces rouxii* groups were lower in fruity odor activity values and were dominated by floral odor. *Hanseniaspora uvarum* J1 and *Hanseniaspora uvarum* J4 treatment groups were dominated by fruity odor, followed by woody and tobacco odor. Cigar leaves treated with *Pichia pastoris* P3 was dominated by fruity odor, followed by floral and woody odor. *Zygosaccharomyces bailii* R6 treated cigar leaves showed higher floral and fruity odor activity values. After yeast inoculation, aroma of cigar filler leaves was improved to various degrees. The aroma of the group inoculated with *Hanseniaspora uvarum* J1 and *Pichia pastoris* P3 was the highest, which were increased by 5.75 times and 5.54 times respectively, compared with the control group sprayed with YEPD.

In terms of overall flavor intensity, the 29 aroma-causing components had higher odor activity values for fruity and floral flavors, followed by tobacco and woody flavors. Presumably because of the lower perceptual thresholds for β-damascenone, dihydrodamascenone, damascenone, and 2-hexenal, which possess both fruit and floral of flavors. Due to the large number of aroma-causing components in tobacco, OAV values for only 29 aroma components were found in this study. Furthermore, cross-influences among aroma-causing components were not considered.

### 3.4 PCA analysis of key aroma components in cigar filler leaves

**FIGURE 3:**
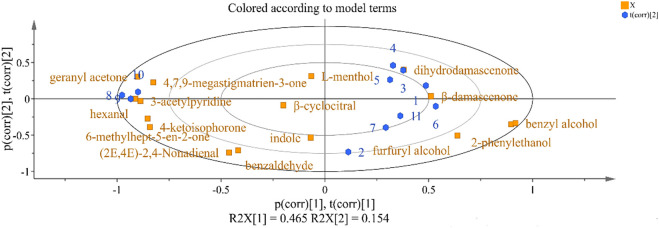
Principal component analysis biplot (score and load values) for volatile compounds in different treatment of fermented Cigar filler leaves. Note: The figures in Figure 3 represent the same groups as in Figure 1.

PCA is a multivariate statistical analysis method, which can be used to analyze databases related to several mutually dependent variables. The aroma components in cigar tobacco leaves are complex and diverse. In order to obtain more accurate and intuitive classification results, 24 volatile components with OAV values greater than 1 in cigar tobacco leaves were used for principal component analysis to observe the difference between cigar tobacco leaves after different treatments. It can be seen from Figure 3 that the variance contribution rates of PC1 and PC2 were 47.41% and 16.86% respectively, which explained 61.9% of the total variance of cigar filler leaves. Most X-variables (OAV of volatile compounds) and Y-variables (different yeast treatments) were around the circle, where *Hanseniaspora uvarum* J1, *Hanseniaspora uvarum* J4, and *Pichia pastoris* P3 were clustered as one category. In addition, *Clavispora lusitaniae*, *Saccharomyces cerevisiae*, and *Cyberlindnera fabianii* were clustered as one category, indicating that there was similarity between the types or concentrations of volatile components in the cigar filler leaves after fermentation. And the treatment group with water only or with medium only was obviously different from other treatment groups.

The correlation between volatile components of cigar filler leaves with different yeasts is indicated by the distance of the principal components on the graph. *Hanseniaspora uvarum* J1, *Hanseniaspora uvarum* J4, and *Pichia pastoris* P3 were mainly closely related to six volatile components, including ketones (geranyl acetone, 4,7,9-methatigmatrien-3-one, 6-methylhept-5-en-2-one, 4-ketoisophone), aldehydes (hexanal), and other types (3-acetylpyridine). *Clavispora lusitaniae*, *Saccharomyces cerevisiae* and *Cyberlindnera fabianii* were mainly related to Damascenone. *ZygoSaccharomyces rouxii* was closely related with β- Damascenone. *Zygosaccharomyces bailii* R6 and *Saccharomycopsis fibuligera* showed great impact on indole, furfuryl alcohol and β- Damascenone formation.

### 3.5 Cluster analysis of volatile components in cigar filler leaves fermented by different yeasts

**FIGURE 4:**
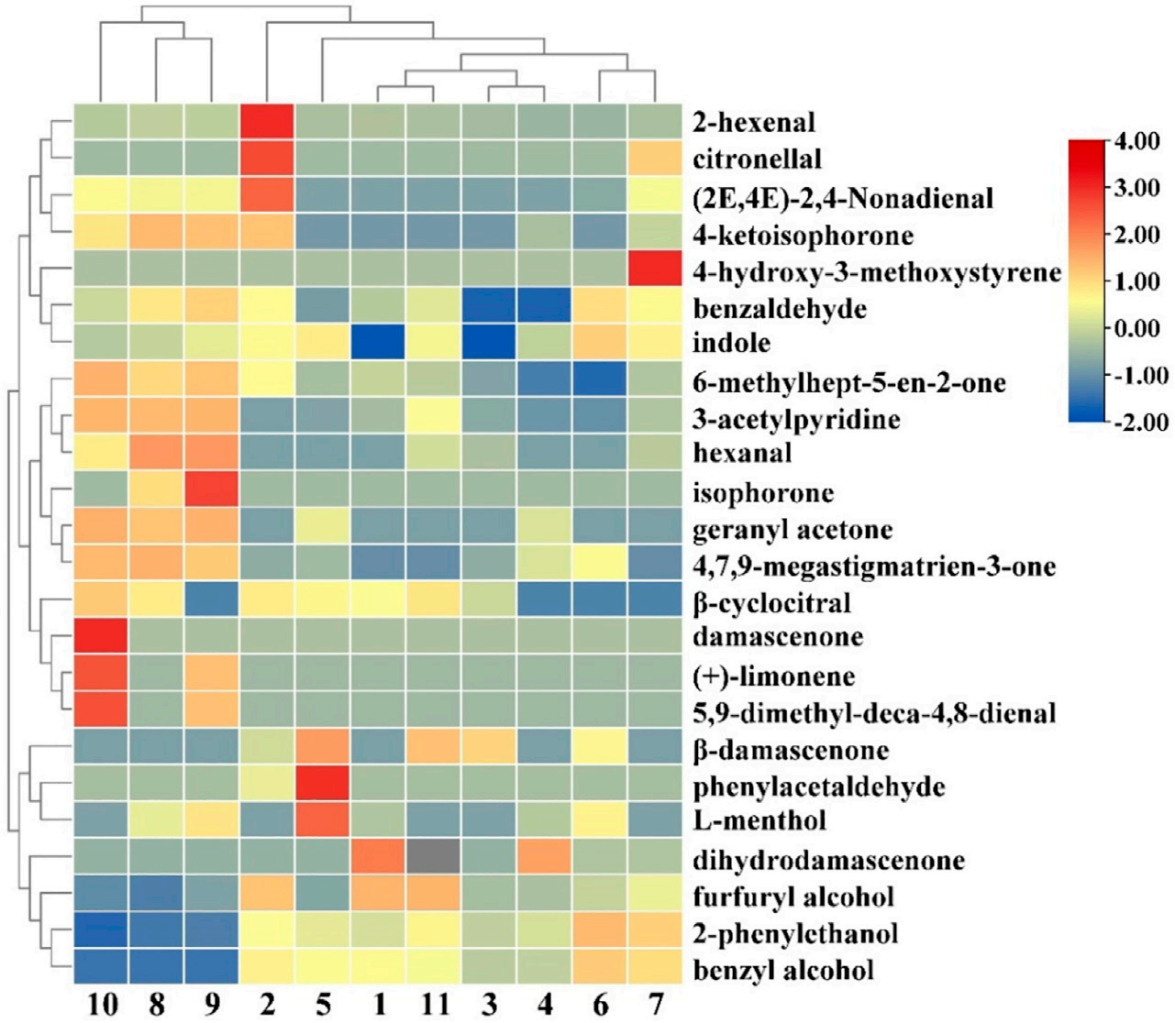
Heat map of cluster analysis of flavor compounds in Cigar filler leaves fermented by different yeasts. Note: The numbers in Figure 4 represent the same groups as in Figure 1.

During cluster analysis, distance between variables was calculated, which indicates the similarity between variables. Cluster analysis results of aroma components of cigar filler leaves fermented by different yeasts were shown in Figure 4. The results showed that there were significant differences among different samples, which was consistent with the PCA analysis results. The volatile components were divided into 3 categories. The first category included 7 compounds, which contained many high content characteristic flavor compounds, including 2-hexenal (pleasant green leaf fragrance and fruit aroma), citronellal (clean herbal citrus odor) and 4-ketoisophorone (strong tea aroma). The second category included 10 kinds of compounds, such as 3-acetylpyridine (sweet, green and earthy smell), geranyl acetone (floral, fruity aroma), and hexanal which not only has the woody aroma, but also has the unique fruit aroma at low concentrations. The third group aggregated seven compounds, such as phenylacetaldehyde (honey, sweet, floral, chocolate and cocoa, with a spice nuance), 2-phenylethanol (floral, sweet, rosy and bready) and furfuryl alcohol (sweet, caramel aroma). Therefore, Category 1 and Category 3 focused on the characteristic aroma compounds of cigar tobacco leaves, ensuring the keynote of cigar tobacco leaves. Category 2 contained the most diverse and dispersed volatile substances, including various styles of aroma substances, reflecting the differences of each sample.

## 4 Discussion

At present, researches on microbial fermentation of tobacco mainly focus on the isolation and screening of strains that degrade nicotine, nitrosamines, macromolecules, and β-carotene. Limited research has been reported on the fermentation of cigar leaves by adding different aroma-producing yeast (Xu et al., 2021). The results of this study showed that the addition of exogenous aroma-producing yeast during artificial fermentation could significantly increase the aroma content of cigar filler leaves, which is basically consistent with the results of Hu et al. (2018). It is possible that the growth and metabolism of aroma-producing yeast during the fermentation of cigar filler leaves promoted the quality improvement of cigar filler leaves.

In recent years, many scholars have made related studies on aroma producing yeast. In this study, nine strains of yeast with good aroma production ability were selected, among which *Clavispora lusitaniae* has been developed for the production of naturally carbonated beverage with improved taste and aroma (Jairath et al., 2012). *Clavispora lusitaniae* isolated from Daqu shows high ability to produce a large amount of ethyl caproate. Furthermore, a total of 30 flavor compounds were detected after fermentation by *Clavispora lusitaniae*, including isobutanol, isoamyl alcohol, furfuralcohol, 3-methylthiopropanol, phenethyl alcohol, phenylethyl acetate, 2-methyl butyric acid-2-ethyl phenyl ester, 2, 4-dimethylbenzaldehyde, 2, 3-dihydrobenzofuran (Fan et al., 2021). Higher alcohols such as furfuralcohol can impart sweetness and enhance the fragrance of other flavor compounds (Luo et al., 2015). Phenethyl alcohol (honey and rose flavors) is an important compound in many fermented products (Lin et al., 2020). The formation of these compounds contributes to the sweet flavor in fermented cigar filler leaves.

During the fermentation of pear wine, a high yield of ester (hexyl acetate, ethyl caprylate and phenylethyl acetate) and alcohols (isoamyl alcohol, hexanol and 2-phenylethanol) compounds were obtained with the help of *Saccharomyces cerevisiae*, which have an important impact on the taste and aroma of pear wine (Yang et al., 2022). Inoculation of *Saccharomyces cerevisiae* to apple wine fermentation can increase the concentration of acetaldehyde and ethyl acetate (He et al., 2022). After adding *Saccharomyces cerevisiae* to fermentation of Chinese wolfberry wine, high content of isoamyl acetate, isoamyl, alcohol propanol, ethyl cinnamate and β-ionone was detected. β-ionone is a representative fruit aroma substance of wolfberry wine, which has a significant impact on the aroma quality of fruit wine (Zhao et al., 2022). *Saccharomyces cerevisiae* plays an extremely important role in koji fermentation. The production capacity of aromatic compounds, alcohols, esters, and acid volatile flavor substances is strong. It shows a high production capacity for 2-phenylethanol, ethyl palmitate, and guaiacol (Xue, 2016). This is consistent with our results, it was indicated that after fermentation of cigar filler leaves by *Saccharomyces cerevisiae*, the content of β-ionone was increased. Furthermore, the contents of β-ionone, solanone, geranyl acetone, 4,7,9-Megastigmatrien-3-one, perhydrofarnesyl acetone, farnesyl acetone, bis(2-ethylhexyl) benzene-1,3-dicarboxylate were increased. Tobacco leaves were fermented with *Saccharomyces cerevisiae* ULI3 and *Maltophilia oligotrophic* earlier. After fermentation, six important aroma components (4,7,9-Megastigmatrien-3-one, Damascone, Solanone, dihydrodamadone, geranyl acetone and dihydroactinidiolide) was increased by 6.65% compared with that before fermentation (Long et al., 2021). Tobacco leaves treated with organic acids and *Saccharomyces cerevisiae* showed that the total water-soluble sugar content and sugar nicotine ratio was increased. And the content of volatile substances such as alcohols, ketones, esters, aldehydes and neophytadiene also increased (Ma C. L et al., 2022). *Saccharomyces cerevisiae* and *Eurotium cristatum* were inoculated with green tea powder. Then the fermentation broth was sprayed to tobacco leaves to make cigarettes. Tea, wine, honey and special flavors were produced after fermentation. The sensory quality of tobacco was improved (Du et al., 2021). In the present study, after inoculation with *Saccharomyces cerevisiae*, the content of ketones and hydrocarbons in volatile compounds of cigar filler leaves was significantly higher than that of the control group with water, which was helpful to improve the aroma quality of tobacco leaves.

The potential benefit of *C. fabianii* is their relatively high levels of ability to produce esters. Among the ester, the most important compounds are ethyl acetate, 3-methylbutyl acetate, methylpropyl acetate, phenylethyl acetate, ethyl hexanoate and ethyl octanoate (Van Rijswijck et al., 2017). The application of *C. fabianii* in rice wine brewing showed that 21 kinds of main flavor compounds were detected, which have a positive effect on increasing the aroma. Isoamyl alcohol and phenethyl alcohol are common flavor compounds in alcoholic drinks. Isoamyl alcohol can impart sweetness and enhance the fragrance of other flavor compound (Jairath et al., 2012), which could only be detected in cigar filler leaves after fermentation with *C. fabiani*.

Previous studies have shown that *ZygoSaccharomyces rouxii* is a osmophilic yeast, which can grow in a high salt, high sugar, high temperature and low pH environment, and can endure a very low water activity environment (Escott et al., 2018; Dai et al., 2020). During soybean sauce fermentation, aromatic compounds such as esters (ethylhexanoate, 2-phenylethylacetate) could be produced to make aroma rich (Han et al., 2020). The concentration of volatile organic compounds in the fermentation process of chilli bean sauce can be significantly increased by *ZygoSaccharomyces rouxii*, and various new volatile organic compounds can be formed, such as 2-phenylethanol, 2-methoxy-phenol and pyrazine (Niu et al., 2022). The content of 2-phenylethanol by inoculation with *ZygoSaccharomyces rouxii* to cigar filler leaves was much higher than that of other groups.

*Saccharomycopsis fibuligera* shows ability to produce aroma and ester. During the fermentation of Chinese rice wine, more 2-phenylethanol, 1-octene-3-ol and 2-octene-1-ol, ethyl acetate, ethyl octanoate, phenylacetaldehyde and ethyl butyrate can be produced. These aromatic substances endow the Chinese rice wine with a comprehensive aroma of flowers, fruits and honey (Lee et al., 2018; Yang et al., 2021). Among the volatile aroma components of solid fermentation products of *Saccharomycopsis fibuligera*, various products also present fruity and floral aromas. The content of ethyl acetate (fruity aroma), isoamyl acetate (banana and pear aroma), 2-phenylethanol (rose aroma), phenethyl acetate (sweet aroma), and ethyl palmitate (fruit and cream aroma) are higher. The sensory evaluation of its solid fermentation products shows a strong fruit flavor, which is the result of these volatile flavor substances (Wang et al., 2017). In this study, the content of ketone compounds (such as solanone, R-(-)-3-Hydroxy-β-ionone, farnesyl acetone) that detected in cigar filler leaves after fermentation by *Saccharomycopsis fibuligera* was the highest.

The contents of methyl caproate, methyl octanoate and methyl caprylate in strawberry fruits fumigated by *Hanseniaspora uvarum* were increased during cold storage (Wang et al., 2019). Farnesol, 2-heptanol, hexan-1-ol, nerol, benzaldehyde, isoamyl acetate, ethyl butanoate, ethyl propionate, phenethyl acetate, linalool, β-damascenone and other aromatic substances produced in the fermentation of grape juice (Ge et al., 2021), make it rich in aroma. In the cigar filler leaves treated by *Hanseniaspora uvarum*, substances such as β-damascenone, 4,7,9-Megastigmatrien-3-one, 4-ketoisophorone, perhydrofarnesyl acetone, farnesyl acetone, 2-hexenal, 4-pyridinecarboxaldehyde, phytol, tributyl phosphate, 3-acetylpyridine were increased.

A large amount of ethyl acetate can be produced by *pichia pastoris* in solid fermentation of millet, showing strong apple flavor (Jia et al., 2021). After inoculation of *pichia pastoris* to distiller’s grains for fermentation, the content of ester was the highest. Among the esters, the content and types of ethyl esters were the highest (Huang et al., 2021). In the present study, the highest content of esters was detected in the group inoculated with *Pichia pastoris*, mainly tribehenin and tributyl phosphate. *Hanseniaspora* sp*.* and *Pichia* sp*.* were employed to investigate the effects on the volatile aroma components of tobacco leaves (Li M et al., 2020). Results showed that a total of 95 volatile aroma components were detected. Among alcohols, benzyl alcohol, phenylethyl alcohol and other important flavor substances were increased, which are the decomposition products of aromatic amino acids, can increase the floral flavor of tobacco leaves. Cigarettes were fermented with three aroma producing strains, including *Bacillus*, *Saccharomyces cerevisiae*, and *Hanseniaspora*. sp. The aroma components of tobacco leaves after solid fermentation was increased to varying degrees compared with the control group. Ketones are the most important neutral aroma components in tobacco and smoke. The highest content of ketones was from the samples treated with *Hanseniaspora*. sp. (Hu et al., 2020). In this study, the nicotine content of *Hanseniaspora uvarum* J1 treated cigar filler leaves was increased after fermentation, and the ratio of nitrogen to nicotine, the ratio of chlorine to potassium were increased, compared with the control group with water. The volatile aroma substances in tobacco leaves fermented by *Hanseniaspora uvarum* J1 and *Hanseniaspora uvarum* J4 were higher than those of the two control groups, and the contents of ketones, aldehydes and neophytadiene were significantly increased, which was similar to the results of Hu et al. (2018).

*Z. Bailii* can produce alcohols, acids, esters, aldehydes, ketones and other flavor substances during Baijiu fermentation, which contributes to the flavor and quality of sauce-flavour Baijiu (Xu M. L et al., 2017). *Z. Bailii* is the dominant strain in the brewing process of sauce-flavour Baijiu. It can produce farnesol, 2-nonanol, 2-ethylhexanol, decanoic acid, lauric acid, octanoic acid and ethyl octanoate. The content of alcohols, lipids and ketones in Baijiu is increased by co-fermentation with *Bacillus licheniformis* (Zhuang et al., 2017). In this study, *Z. Bailii* R6 was inoculated to cigar filler leaves, and substances with higher content compared with other yeasts were β-Damascenone, 6-nonenal and 3-methylthiopropanal.

After yeast addition, the total amount of volatile substances in the cigar leaves was increased after fermentation. And cigar leaves fermented by *ZygoSaccharomyces rouxii* shows the highest content of esters. As one of the important flavor substances, easter is a class of volatile compounds with aromatic odors that act as a flavor enhancer and flavoring agent in tobacco (Ruan et al., 2006) and can improve the smoking quality of the tobacco. After fermentation of cigar filler leaves, the increase of some substances can reduce the irritation of tobacco leaves, such as solanone, geranyl acetone, dihydroactinidiolide, megalenone, neophytadiene, etc. The representative substances of aromatic compounds are mainly benzyl alcohol, phenylethanol, benzaldehyde, phenylacetaldehyde, etc., which can improve concentration of the smoke, and play a greater role in the aroma characteristics (Zhang P et al., 2021; Ma X. W et al., 2022).

The fermentation of tobacco bud by aroma producing yeast and Maillard reaction was studied by Xu Y et al. (2018). The results showed that there were 63 kinds of aroma substances, among which palmitic acid, tetradecanoic acid, palmitoleic acid, benzyl alcohol, 2-phenylethanol, fitone were in rich amount. 4,7,9-Megastigmatrin-3-one, dihydroactinidiolide, oleamide and 2-pentylfuran make outstanding contributions to tobacco aroma. Aroma producing yeast were screened and applied to treat low grade tobacco leaves. Relative contents of aroma components were changed greatly, including phenylethanol, benzyl alcohol, 1-benzofuran, heptadecane, 4,7,9-Megastigmatrien-3-one, palmitic acid, geranyl acetone, geraniol and other alcohols and ketones. Furthermore, the content of aldehydes (pentanal, crotonaldehyde) in low grade tobacco leaves after microbial treatment was significantly reduced, which often caused smoking irritation (Chen, 2013).

PCA has been widely applied in the analysis of main aroma components in food industry (Wang et al., 2016; Xu Y et al., 2017). By PCA, it was concluded that acetic acid, acetoin, 1-octen-3-one, and 3-methylindole are the most important compounds that lead to the difference between mild cheddar cheese and other medium and mature cheddar cheese (Wang et al., 2021). Our analysis results showed that *Hanseniaspora uvarum* J1, *Hanseniaspora uvarum* J4, and *Pichia pastoris* P3 were clustered as one category. *Clavispora lusitaniae*, *Saccharomyces cerevisiae*, and *Cyberlindnera fabianii* were clustered as one category. Because the volatile compounds were formed by biological and chemical transformations, it was concluded that these typical aroma compounds, which made one category different from the other, were largely influenced by the fermentation with different yeasts.

## 5 Conclusion

Nine strains of aroma-producing yeast with good liquid fermentation were selected for artificial solid-state fermentation, and the aroma-causing components of the fermented cigar leaves were analyzed and compared. A total number of 52 aroma compounds contributed to the flavor of cigar in all treatment groups, among which β-damascenone, dihydrodamascenone, damascenone, and 2-hexenal contributed the most. By comparing the odor activity values, it was found that cigar leaves treated with *Clavispora lusitaniae*, *Cyberlindnera fabianii*, and *Saccharomycopsis fibuligera* had the highest floral odor activity values. The highest fruity odor activity values were found in cigar leaves treated with *Hanseniaspora uvarum* J1, *Hanseniaspora uvarum* J4 and *Pichia pastoris* P3. It was found that yeast addition could make the aroma richer and reduce the original impurities and irritation of the cigar leaves. With the rapid development of biological research, the application of microbiology in tobacco leaves fermentation is of great significance to improve the smoking quality of cigarettes.

## Data Availability

The raw data supporting the conclusions of this article will be made available by the authors, without undue reservation.
