# Supplementary material for: Application of yeast in plant-derived aroma formation from cigar filler leaves
Source: Front Bioeng Biotechnol. 2022 Dec 21;10:1093755. doi: 10.3389/fbioe.2022.1093755 (PMC9815610; doi:10.3389/fbioe.2022.1093755)
Supplement: Supplementary file 1 [file Table1.PDF]

Table S1 Contents of volatile components in cigar filler leaves fermented by different yeasts

Category	number	chemical composition	1	2	3	4	5	6	7	8	9	10	11
	1	2-cyclopentylcyclopentanone	0.50	-	-	-	0.26	0.35	0.24	-	-	-	-
	2	2-sec-Butylcyclopentanone	0.29	0.44	-	0.04	0.26	0.08	0.15	-	0.88	0.66	0.40
	3	1,1,10-trimethyl-trans-decalone	-	-	-	-	-	-	0.01	0.03	-	-	-
	4	6-methyloct-5-en-2-one	0.12	0.52	0.03	-	-	-	0.23	-	-	0.21	0.07
	5	6-methylhept-5-en-2-one	1.62	2.23	0.94	0.41	1.31	0.13	1.43	2.63	2.89	3.07	1.49
	6	3,6-dimethyloctan-2-one	-	-	-	-	0.17	-	0.23	-	-	-	0.31
	7	solanone	12.76	11.37	15.81	14.31	22.69	-	24.14	-	-	-	17.83
	8	4-(4-hydroxy-2,6,6-trimethylcyclohexen-1-yl)but-3-en-2-one	-	-	2.52	-	-	-	0.85	-	-	-	0.59
	9	neryl acetone	-	-	2.39	-	-	-	-	-	-	-	-
	10	geranyl acetone	-	-	-	2.23	2.62	-	-	4.66	5.12	5.23	-
Ketones	11	4,7,9-Megastigmatrien-3-one	-	5.05	5.14	14.37	7.21	18.34	-	29.06	25.66	28.00	-
	12	3-ethoxy-2-methylcyclopent-2-en-1-one	-	-	-	0.17	-	-	-	-	-	0.24	-
	13	isophorone	-	-	-	-	-	-	-	0.27	0.61	-	-
	14	2,2,6-trimethyl-1,4-cyclohexanedione	-	-	-	-	-	-	-	1.99	1.92	-	-
	15	4-ketoisophorone	-	2.59	-	0.66	-	-	1.02	2.72	2.60	2.10	-
	16	perhydrofarnesyl Acetone	11.84	14.68	7.87	12.79	18.92	8.93	17.20	18.09	14.73	18.54	12.75
	17	farnesyl acetone	11.53	10.65	8.43	12.99	18.41	9.71	18.36	18.83	21.32	15.89	13.11
	18	β-damascenone	-	2.75	5.94	-	8.03	4.57	-	-	-	-	6.63
	19	dihydrodamascenone	-	-	-	-	-	-	-	-	-	1.78	-
	20	damascenone	7.40	-	-	6.07	-	0.79	8.26	-	-	-	-
		Ketones totals	46.06	50.28	49.07	64.04	79.88	42.90	72.12	78.28	75.73	75.72	53.18

Category	number	chemical composition	1	2	3	4	5	6	7	8	9	10	11
	21	hexanal	-	-	0.47	-	-	-	0.63	2.60	2.59	1.59	0.93
	22	furfural	-	1.32	0.52	-	7.18	0.87	1.18	-	-	-	1.51
	23	6-nonenal	-	-	-	-	-	-	-	-	-	-	0.11
	24	2-hexenal	0.98	1.86	0.48	-	0.72	-	0.72	2.03	1.92	1.51	0.87
	25	pyridine-3-carbaldehyde	-	-	0.63	-	-	-	-	-	-	-	-
	26	3-methylthiopropanal	-	0.59	-	-	-	-	-	-	-	0.15	0.31
	27	phenylacetaldehyde	-	1.31	-	-	6.03	-	-	-	-	-	-
	28	benzaldehyde	0.88	1.07	0.50	0.50	0.71	1.16	1.08	1.13	1.20	0.93	0.99
	29	5-methyl furfural	-	0.85	-	0.65	-	2.58	1.03	-	-	-	0.56
	30	(2E,4E)-2,4-Nonadienal	-	1.52	-	-	-	0.04	0.62	0.60	0.60	0.67	-
	31	4-pyridinecarboxaldehyde	0.92	1.24	-	-	0.60	1.21	-	1.27	1.36	1.06	1.42
	32	tetradec-13-enal	-	-	-	-	-	-	0.06	-	0.06	-	0.07
Aldehydes	33	1-methyl-1H-pyrazole-4-carbaldehyde	0.31	-	0.28	-	0.51	-	-	-	-	-	-
	34	(Z)-7-hexadecenal	0.51	0.61	0.94	0.08	0.42	0.15	0.17	0.33	0.16	1.26	-
	35	2-ethylidene-6-methylhepta-3,5-dienal	-	-	0.28	-	-	-	0.39	-	0.25	-	0.45
	36	citronellal	-	1.11	-	-	-	-	0.56	-	-	-	-
	37	β-cyclocitral	0.23	0.26	0.16	-	0.24	-	-	0.26	-	0.31	0.27
	38	farnesal	1.16	-	0.89	1.05	1.29	-	1.80	-	-	-	1.55
	39	all-trans-retinal	3.44	2.58	0.34	1.44	4.29	0.76	2.88	-	0.17	-	-
	40	5,9-dimethyl-deca-4,8-dienal	-	-	-	-	-	-	-	-	0.08	0.14	-
	41	3-methylcyclohex-3-ene-1-carbaldehyde	-	-	-	-	0.23	0.12	-	-	-	-	-
	42	3-formyl-5,5-dimethyl-2-cycolohexen-1-one	-	-	-	-	0.91	-	-	-	-	-	-
	43	3-(2,6,6-trimethyl-1-cyclohexen-1-yl)acrylaldehyde	3.30	1.68	2.75	3.55	3.33	-	3.42	-	-	-	3.21
	44	4-methyl-2-(1-methylethyl)-2-hexenal	-	-	-	-	0.43	-	-	-	-	-	-
	45	A ,2,6,6-tetramethyl-1-cyclohexene-1-crotonaldehyde	8.24	5.58	2.29	3.99	2.54	2.78	8.00	-	4.18	3.21	4.85
		Aldehydes totals	19.97	21.58	10.53	11.26	29.43	9.67	22.54	8.22	12.57	10.83	17.10

Category	number	chemical composition	1	2	3	4	5	6	7	8	9	10	11
Alcohols	46	furfuryl alcohol	6.83	6.35	2.27	2.38	1.38	3.02	4.16	-	1.28	0.45	6.82
	47	(2,5-dimethyl-3,4-dihydropyran-2-yl)methanol	-	-	-	-	-	-	0.71	-	-	-	-
	48	L-menthol	0.09	-	-	0.10	0.54	0.25	-	0.19	0.28	-	-
	49	6-ethyl-3-methyloct-5-en-1-ol	-	-	-	-	-	0.05	-	-	-	-	-
	50	(s)-cis-verbenol	0.11	0.21	0.07	-	0.43	-	0.19	-	-	-	0.15
	51	benzyl alcohol	5.03	5.23	3.12	3.28	4.83	6.33	5.89	-	-	-	4.76
	52	2-phenylethanol	4.13	5.03	3.70	4.16	4.48	6.66	6.15	1.09	1.26	0.55	5.30
	53	3-benzyloxy-1-propanol	-	-	-	0.18	-	-	-	-	-	-	-
	54	bicyclo[2.2.2]octane-1,4-diyldimethanol	-	-	0.36	-	0.40	-	-	-	-	0.14	-
	55	2-methylene-5α-cholestan-3β-ol	1.42	2.77	2.41	-	6.29	2.77	2.81	-	7.15	6.97	1.50
	56	3,7,11,15-tetramethylhexadec-1-yn-3-ol	-	-	-	-	-	0.21	0.34	-	-	-	4.31
	57	3,7,11-trimethyldodecan-1-ol	-	-	-	-	0.41	-	0.34	-	-	-	-
	58	1,2,3,4-tetrahydro-2,5,8-trimethylnaphthalen-1-ol	3.78	-	-	-	3.84	3.53	1.15	-	-	-	-
	59	2,5-dimethylhexa-1,5-diene-3,4-diol	-	-	-	0.37	-	-	-	0.26	-	-	-
	60	cis/trans-p-menthadienol	-	-	-	-	-	0.19	0.57	-	-	-	-
	61	isoamyl alcohol	-	-	-	-	0.03	-	-	-	-	-	-
	62	(2,2,6-trimethylbicyclo[4.1.0]hept-1-yl)methanol	-	2.66	4.46	-	5.30	-	1.71	-	-	-	-
	63	6-ethyl-3-methyloct-5-en-1-ol	-	-	-	-	-	0.05	-	-	-	-	-
	64	dihydromyrcenol	-	-	-	-	-	-	-	-	-	0.05	-
	65	trans--terpineol	-	-	0.31	-	-	-	-	-	-	-	-
	66	isopinocarveol	-	-	0.78	-	-	-	-	0.19	-	-	-
	67	isopulegol	-	-	-	-	-	-	-	0.24	-	-	-
	68	carotatoxin	-	-	-	0.12	-	-	0.34	-	-	-	-
	69	phytol	60.27	80.84	56.45	57.54	130.73	36.71	123.94	122.66	120.88	65.69	100.46
		Alcohols totals	81.66	103.09	73.93	68.13	158.66	59.77	148.30	124.63	130.85	73.85	123.30

Category	number	chemical composition	1	2	3	4	5	6	7	8	9	10	11
Esters	70	(E)-2-octenyl 2-methyl-(E)-2-butenoate	-	-	-	0.60	-	-	-	0.10	-	-	0.46
	71	dodecan-2-yl 2,2,2-trifluoroacetate	-	-	-	-	-	-	0.12	-	-	-	-
	72	3-methylcyclopentyl acetate	-	-	1.83	-	2.76	-	4.42	2.35	-	-	-
	73	12,15-octadecadiynoic acid methyl ester	-	-	-	-	-	-	0.26	-	-	-	-
	74	geranyl isovalerate	0.12	1.91	-	0.11	-	0.35	0.32	-	1.93	-	-
	75	bis(2-ethylhexyl) isophthalate	-	-	12.10	31.29	-	-	-	-	-	-	-
	76	2,5-octadecadiynoic acid methyl ester	-	-	-	-	-	0.09	0.25	-	-	-	-
	77	tribehenin	-	-	-	-	-	-	-	-	-	8.41	-
	78	1-α-linolenoylglycerol	-	-	-	-	-	1.73	-	-	-	-	-
	79	α-pinene oxide	-	-	-	-	-	-	-	-	0.07	-	-
	80	tributyl phosphate	-	-	-	-	-	3.90	-	11.77	9.42	11.67	-
		Esters totals	0.12	1.91	13.93	32.00	2.76	6.07	5.37	14.22	11.42	20.08	0.46
Hydrocarbons	81	(+)-Limonene	-	-	-	-	-	-	-	-	0.31	0.53	-
	82	3-(2-methylpropyl)-1-cyclohexene	-	-	0.35	-	-	-	-	-	-	-	-
	83	7-tetradecene	-	-	-	-	-	-	0.08	-	-	-	-
	84	ethyl linalyl ether	-	-	-	-	-	-	0.14	-	-	-	-
	85	6-methylenespiro[4.5]decane	-	-	-	-	-	-	0.21	-	-	-	-
	86	1,2-epoxycycloheptane	-	-	-	-	0.02	-	-	-	0.05	-	0.05
	87	4-methyldocosane	-	-	-	-	-	-	-	-	-	-	0.07
	88	octane, 1-ethoxy	-	-	-	-	-	-	-	-	-	-	0.03
	89	3-buten-1-ynyltrimethylsilane	-	-	-	-	-	-	0.40	-	-	-	-
	90	6-methyloctadecane	-	-	-	0.54	-	0.27	0.16	-	0.33	-	-
	91	dipentene dioxide	1.49	1.40	1.45	-	6.37	-	0.57	0.27	3.43	0.16	-
	92	neophytadiene	149.70	107.04	113.99	175.33	317.15	216.31	270.36	206.17	240.04	153.77	146.83
		Hydrocarbons totals	151.19	108.44	115.79	175.87	323.54	216.58	271.92	206.44	244.16	154.46	146.98

Category	number	chemical composition	1	2	3	4	5	6	7	8	9	10	11
Acids	93	dec-3-ynoic acid	-	-	-	-	0.86	-	-	-	-	-	-
	94	3-hydroxylauric acid	-	0.74	-	-	-	-	-	-	-	0.21	-
	95	nervonoyl acid	-	-	0.44	-	-	-	-	-	-	0.34	0.51
	96	paullinic acid	-	-	-	-	-	-	-	0.35	2.19	-	-
		Acids totals	0.00	0.74	0.44	0.00	0.86	0.00	0.00	0.35	2.19	0.55	0.51
Others	97	indole	7.53	7.96	-	5.74	8.45	9.59	8.22	5.87	6.96	5.32	7.48
	98	3-acetylpyridine	3.28	2.02	2.43	1.62	2.20	1.39	3.65	8.40	8.57	8.53	6.03
	99	2,3'-bipyridine	16.02	-	22.24	12.34	19.82	11.14	18.98	29.40	11.14	33.40	33.47
	100	prop-2-enoxymethylbenzene	-	-	-	-	-	-	6.97	-	-	-	5.98
	101	o-Xylene	-	-	0.87	-	3.64	-	0.72	-	-	-	-
	102	benzenepropanamide	-	-	-	-	8.09	-	-	-	-	-	1.56
	103	4-hydroxy-3-methoxystyrene	-	-	-	-	-	-	2.36	-	-	-	-
	104	1,4,6-trimethyl-5,6-dihydronaphthalene	-	-	-	-	-	-	1.94	-	-	-	-
	105	caryophyllene oxide	0.10	-	-	-	-	-	-	-	0.13	-	-
	106	pyrrole	-	-	-	-	-	-	1.02	-	-	-	-
	107	(Z)-verbenol	0.11	-	0.07	0.21	-	-	-	-	-	-	-
	108	isothujol	-	-	-	0.08	-	-	-	-	-	-	-
	109	indolizine	-	-	6.18	-	-	-	-	-	-	-	-
	110	o-nicotine	-	-	-	-	-	-	-	-	9.30	-	-
	111	2-methyl-6-quinolinamine	-	-	-	-	-	-	-	-	-	16.97	-
	112	ethyl linalyl ether	-	-	-	-	-	-	-	-	-	0.33	-
	113	cis-carvone oxide	-	-	-	-	-	-	-	4.03	-	-	-
	114	(E)-3-Pyridinecarbaldehyde o-acetyl oxime	-	-	-	-	-	-	0.12	0.07	0.06	0.06	-
	115	camphor	-	-	-	-	-	0.02	-	-	-	-	0.03
	116	5-hydroxypyrimidine	0.07	-	-	-	0.05	0.04	0.11	-	-	-	0.07
	117	4-methylpyrimidine	-	-	-	-	-	0.15	-	-	-	-	-
	118	o-benzyl-L-serine	-	-	3.81	3.48	-	-	-	-	-	-	-
	119	2,2'-methylenebis(6-tert-butyl-4-methylphenol)	-	-	12.39	59.45	-	-	-	-	-	-	26.60
		Others totals	27.11	9.98	47.99	82.92	42.25	22.33	44.08	47.77	36.16	64.61	81.22
		total	326.11	296.02	311.68	434.22	637.38	357.32	564.33	479.91	513.08	400.10	422.75
		Total amount (excluding neophydiene)	176.41	188.98	197.69	258.89	320.23	141.01	293.97	273.74	273.04	246.33	275.92

Table S2. OAV values of aromatic components in cigar filler leaves after different fermentation

scent	compound	threshold (ug/g)	OAV value										
			1	2	3	4	5	6	7	8	9	10	11
Baked	furfural	9.56	-	0.14	0.05	-	0.75	0.09	0.12	-	-	-	0.16
	5-methyl furfural	22.00	-	0.04	-	0.03	-	0.12	0.05	-	-	-	0.03
	furfuryl alcohol	0.18	37.94	35.28	12.61	13.22	7.67	16.78	23.11	-	7.11	2.50	37.89
	4-hydroxy-3-methoxystyrene	0.01	-	-	-	-	-	-	196.67	-	-	-	-
	3-acetylpyridine	0.50	6.56	4.04	4.86	3.24	4.40	2.78	7.30	16.80	17.14	17.06	12.06
	pyrrole	20.00	-	-	-	-	-	-	0.05	-	-	-	-
	Total		44.50	39.49	17.53	16.49	12.82	19.77	227.30	16.80	24.25	19.56	50.13
fruit	6-methylhept-5-en-2-one	0.07	23.82	32.79	13.82	6.03	19.26	1.91	21.03	38.68	42.50	45.15	21.91
	β-cyclocitral	0.003	76.67	86.67	53.33	-	80.00	-	-	86.67	-	103.33	90.00
	(+)-limonene	0.01	-	-	-	-	-	-	-	-	31.00	53.00	-
	5,9-dimethyl-deca-4,8-dienal	0.00007	-	-	-	-	-	-	-	-	1142.86	2000.00	-
	2-hexenal	0.00002	57647.06	109411.76	28235.29	-	42352.94	-	42352.94	119411.76	112941.18	88823.53	51176.47
	isoamylol	2.80	-	-	-	-	0.01	-	-	-	-	-	-
	benzaldehyde	0.75	1.17	1.43	0.67	0.67	0.95	1.55	1.44	1.51	1.60	1.24	1.32
	Total		57748.72	109532.65	28303.12	6.70	42453.16	3.46	42375.41	119538.61	114159.13	91026.25	51289.70
flowers	dihydrodamascenone	0.000002	3700000.00	-	-	3035000.00	-	395000.00	4130000.00	-	-	-	-
	damascenone	0.00009	-	-	-	-	-	-	-	-	-	19777.78	-
	β-damascenone	0.000002	-	1375000.00	2970000.00	-	4015000.00	2285000.00	-	-	-	-	3315000.00
	(2E,4E)-2,4-Nonadienal	0.06	-	26.67	-	-	-	0.70	10.88	10.53	10.53	11.75	-
	citronellal	0.01	-	185.00	-	-	-	-	93.33	-	-	-	-
	2-phenylethanol	0.36	11.47	13.97	10.28	11.56	12.44	18.50	17.08	3.03	3.50	1.53	14.72
	indole	0.01	684.55	723.64	-	521.82	768.18	871.82	747.27	533.64	632.73	483.64	680.00
	benzyl alcohol	2.55	1.98	2.05	1.23	1.29	1.90	2.49	2.31	-	-	-	1.87
	phenylacetaldehyde	0.01	-	207.94	-	-	957.14	-	-	-	-	-	-
	geranyl acetone	0.06	-	-	-	37.17	43.67	-	-	77.67	85.33	87.17	-
	Total		3700697.99	1376159.27	2970011.50	3035571.83	4016783.33	2680893.51	4130870.88	624.86	732.09	20361.86	3315696.59
Tobacco	4,7,9-megastigmatrien-3-one	0.00386	-	1308.29	1331.61	3722.80	1867.88	4751.30	-	7528.50	6647.67	7253.89	-
Woody	isophorone	0.48	-	-	-	-	-	-	-	0.56	1.27	-	-
	4-ketoisophorone	1.25	-	2.07	-	0.53	-	-	0.82	2.18	2.08	1.68	-
	hexanal	0.001	-	-	470.00	-	-	-	630.00	2600.00	2590.00	1590.00	930.00

	Total		0.00	1310.36	1801.61	3723.33	1867.88	4751.30	630.82	10131.24	9241.02	8845.57	930.00

scent	compound	threshold (ug/g)	OVA										
			1	2	3	4	5	6	7	8	9	10	11
Herb	L-menthol	0.10	0.90	-	-	1.00	5.40	2.50	-	1.90	2.80	-	-
	trans--terpineol	0.35	-	-	0.89	-	-	-	-	-	-	-	-
	Total		0.90	0.00	0.89	1.00	5.40	2.50	0.00	1.90	2.80	0.00	0.00
